# Greater locus coeruleus integrity predicts less accumulation of plasma pTau231 and GFAP over time

**DOI:** 10.1002/alz.093277

**Published:** 2025-01-09

**Authors:** McKenna E. Williams, Christine Fennema‐Notestine, Tyler R. Bell, Carol E. Franz, Stephen J. Glatt, Matthew S. Panizzon, Robert A. Rissman, William S. Kremen, Jeremy A. Elman

**Affiliations:** ^1^ SDSU/UC San Diego Joint Doctoral Program in Clinical Psychology, San Diego, CA USA; ^2^ University of California, San Diego, La Jolla, CA USA; ^3^ SUNY Upstate Medical University, Syracuse, NY USA

## Abstract

**Background:**

The locus coeruleus (LC) is one of the earliest sites of tau accumulation and integrity of the rostral‐middle LC may be a particularly early marker of AD‐related changes. LC dysfunction may also promote further pathological accumulation. We examined relationships between LC integrity and plasma phospho‐tau (pTau) and the astrocytic marker glial fibrillary acidic protein (GFAP), and whether baseline LC integrity moderates subsequent change in biomarker levels.

**Method:**

Participants were 669 men from waves 3 and 4 of the Vietnam Era Twin Study of Aging (VETSA) (wave 3 age=67.423 [SD=2.59], follow‐up interval=5.52 years [SD=0.39]). All were cognitively unimpaired at wave 3. Linear mixed effects models tested concurrent and longitudinal relationships between baseline MRI‐assessed LC rostral‐middle integrity and pTau231 and GFAP across level of genetic risk for AD (APOE‐ε4+ versus APOE‐ε4‐). Covariates included storage time, testing site, and age.

**Result:**

Age was positively associated with pTau231 (β=0.11, p=0.024) and GFAP levels (β=0.26, p<0.001). APOE‐ε4+ carriers showed higher levels of GFAP compared non‐carriers (β=0.23, p=0.011); APOE‐ε4 groups did not differ in levels of pTau231 or LC integrity. Baseline LC integrity was not associated with concurrent biomarker levels in either APOE‐ε4 group. In longitudinal analyses, pTau231 and GFAP increased from waves 3 to 4 (β=0.94, p=0.006, β=1.23, p<0.001, respectively). However, baseline LC integrity moderated the rate of increases, such that higher LC integrity at wave 3 was associated with less pTau231 accumulation (interaction β=‐0.19, p=0.027) for both APOE‐ε4 groups. Additionally, baseline LC integrity moderated change in GFAP from waves 3‐4, and this effect significantly differed by APOE‐ε4 status (3‐way interaction β=‐0.38, p=0.004). Effects of baseline LC integrity on biomarker change were specific to rostral‐middle but not caudal regions. Wave 3 biomarker levels did not moderate longitudinal change in LC integrity.

**Conclusion:**

Results support the idea that higher baseline rostral‐middle LC integrity is protective against factors that cause accumulation of pTau231 and GFAP. However, the nature of the moderating effect of LC integrity on change in GFAP may be dependent on APOE‐ε4 status. Baseline biomarker levels did not moderate change in LC integrity over time, suggesting changes in LC may be an upstream event.

